# Genetic and clinical features of hereditary transthyretin amyloidosis: a decade of experience at a Japanese referral center

**DOI:** 10.1186/s13023-025-04006-6

**Published:** 2025-09-02

**Authors:** Toshiya Nomura, Yohei Misumi, Masayoshi Tasaki, Shiori Yamakawa, Tomoaki Taguchi, Konen Obayashi, Taro Yamashita, Yukio Ando, Mitsuharu Ueda

**Affiliations:** 1 Department of Neurology, Graduate School of Medical Sciences, Kumamoto University, 1-1-1 Honjo, Kumamoto, 860-0811 Japan; 2 Amyloidosis center, Kumamoto University Hospital, Kumamoto, Japan; 3 Department of Clinical Biosciences, Graduate School of Health Sciences, Kumamoto University, Kumamoto, Japan; 4 Department of Morphological and Physiological Sciences, Graduate School of Health Sciences, Kumamoto University, Kumamoto, Japan; 5 Department of Neurology, Soyo Hospital, Kumamoto, Japan; 6 Department of Amyloidosis Research, Nagasaki International University, Nagasaki, Japan; 7 Department of Amyloidosis Supporting Center, Sugimura Hospital, Kumamoto, Japan

**Keywords:** Amyloid, Transthyretin, Hereditary transthyretin amyloidosis, Endemic areas, Non-endemic areas

## Abstract

**Background:**

Hereditary transthyretin (ATTRv) amyloidosis is a rare, intractable genetic disorder caused by mutations in the transthyretin (*TTR*) gene. More than 150 *TTR* mutations have been identified, along with genotype-phenotype correlations. Early diagnosis is critical to facilitate the timely initiation of disease-modifying therapies.

**Objective:**

To characterize the genetic and clinical features of ATTRv amyloidosis at our referral center.

**Methods:**

A total of 6,201 *TTR* genetic tests were conducted at Kumamoto University Hospital over a ten-year period and revealed 289 mutations, including 235 symptomatic cases, which were analyzed in this study.

**Results:**

In a cohort of 235 patients with symptomatic ATTRv amyloidosis, 46 *TTR* mutations were identified. The genotypes were distributed as follows: V30M in endemic areas (7.7%), V30M in non-endemic areas (48.5%), and non-V30M mutations (43.8%). The mean age of onset was lowest for patients with V30M in endemic areas (42.4 ± 15.6 years) and higher for those with non-V30M mutations (60.6 ± 14.6 years) and V30M in non-endemic areas (64.5 ± 11.9 years). Family history was present in 93.3% of V30M cases in endemic areas but absent in 42.0% of V30M cases in non-endemic areas and 57.5% of non-V30M cases. Polyneuropathy was the predominant initial symptom, affecting 73.7% of endemic V30M cases, 54.3% of non-endemic V30M cases, and 34.6% of non-V30M cases. Diagnosis occurred earlier in patients with V30M in endemic areas than in other groups. Notably, delayed diagnosis has been observed in patients presenting with carpal tunnel syndrome or polyneuropathy.

**Conclusions:**

These findings demonstrate that patients with V30M in non-endemic areas and those with non-V30M mutations are more prevalent than previously recognized and that their genetic and clinical characteristics exhibit considerable diversity.

## Introduction

Hereditary transthyretin (ATTRv) amyloidosis is a rare and fatal genetic disorder caused by mutations in the transthyretin (*TTR*) gene. The disease is characterized by a wide spectrum of clinical manifestations, including sensorimotor polyneuropathy, autonomic dysfunction, cardiomyopathy, and gastrointestinal symptoms [1]. First described by Andrade in Portugal, subsequent cases were identified in Japan and Sweden, with the V30M mutation in *TTR* being a prevalent finding in these endemic regions [2–5]. Although once thought to be restricted to endemic areas such as Portugal, Japan, and Sweden, ATTRv amyloidosis is now known to occur globally, with more than 150 pathogenic *TTR* mutations reported [6, 7].

Genotype-phenotype correlations have provided insights into disease variability. The V30M mutation, the most frequently identified mutation, exhibits distinct geographic differences in age of onset and penetrance between endemic and non-endemic regions [8, 9]. Non-V30M mutations are associated with various phenotypes, including neuropathy-dominant, cardiomyopathy-dominant, mixed phenotypes, and oculoleptomeningeal-dominant forms [10].

Recent advancements in disease-modifying therapies, such as liver transplantation, and novel pharmacological interventions, such as TTR tetramer stabilizers and *TTR* gene silencing therapies, have significantly improved the prognosis and quality of life of patients [11–17]. Nevertheless, phenotypic heterogeneity continues to pose challenges for early diagnosis, which is crucial for effective treatment.

To elucidate genotype-phenotype variations, this study analyzed the genetic and clinical profiles of patients with ATTRv amyloidosis diagnosed at the Amyloidosis Center, Kumamoto University Hospital.

## Patients and methods

### Patients

A total of 6,201 *TTR* genetic tests were performed on individuals at the Amyloidosis Center, Kumamoto University Hospital, from April 1, 2013, to March 31, 2023. The cohort included individuals suspected of having ATTRv amyloidosis or requiring differentiation from ATTRwt amyloidosis among those diagnosed with ATTR amyloidosis. *TTR* mutations were detected in 289 individuals, of whom 235 were symptomatic, and 54 were asymptomatic carriers. This retrospective study focused on the clinical and genetic characteristics of 235 patients with symptomatic ATTRv amyloidosis.

### Methods

This study examined the clinical data of 235 patients with ATTRv amyloidosis, focusing on mutation types, patient numbers, geographic distribution by variant, age at diagnosis, sex, initial symptoms, time from onset to diagnosis, endemic versus non-endemic origins, and family history.

Additionally, this study analyzed blood data, including serum M-protein and serum free light chains (FLCs), as well as image data, including 99mTc-PYP scintigraphy, based on the request forms received from 2020 to 2023. Myocardial PYP uptake was assessed using a semi-quantitative visual grading system (Grade 0: no uptake and normal bone uptake; Grade 1: uptake less than rib uptake; Grade 2: uptake equal to rib uptake; Grade 3: uptake greater than rib uptake with mild or absent rib uptake). Grade 2 or 3 myocardial uptake was defined as 99mTc-PYP-positive uptake in the heart [18]. TTR gene analysis was performed according to previously established protocols [19]. This study also assessed the detection rate of ATTR amyloid deposits in biopsied tissue samples obtained from 210 patients. Pathological evaluations included Congo red staining and immunostaining with antibodies specific to amyloid A, AL κ (κ 116–133), AL λ (λ 118–134), and TTR 115–124, as described in previous studies [19].

### Statistical analysis

Descriptive analyses were conducted for patient characteristics, with categorical data summarized as numbers and proportions (%) and continuous data presented as means with standard deviations (SD). All statistical analyses and graphical representations in this study were performed using Microsoft Excel 2021 (Microsoft Corporation, Redmond, WA, USA), the “meta” package in R Statistical Software (version 4.1.3), and RStudio.

## Results

**Fig. 1:**
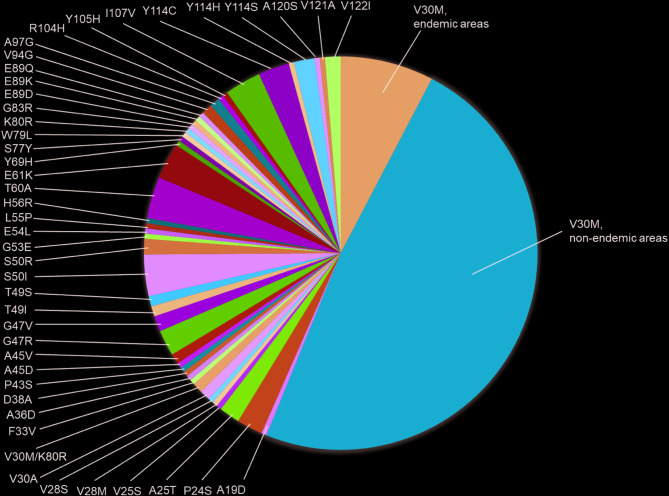
Mutation landscape in ATTRv amyloidosis. This figure illustrates the distribution of *TTR* mutations in 235 patients with symptomatic ATTRv amyloidosis. The analysis highlights the relative proportions of V30M mutations in endemic and non-endemic areas and non-V30M mutations, emphasizing the genetic diversity of the cohort

In this study, 46 *TTR* gene mutations were identified in 235 symptomatic ATTRv amyloidosis patients, including V30M, A19D, P24S, S50I, and T60A mutations (Fig. 1). V30M was the most common mutation, present in 132 patients (56.2%), predominantly in non-endemic areas (86.4%) compared to endemic regions (13.6%). Non-V30M mutations accounted for 43.8% of the cases (*n* = 103), with S50I and T60A being the most frequently observed variants. These findings highlight the extensive genetic variability associated with ATTRv amyloidosis.

**Fig. 2:**
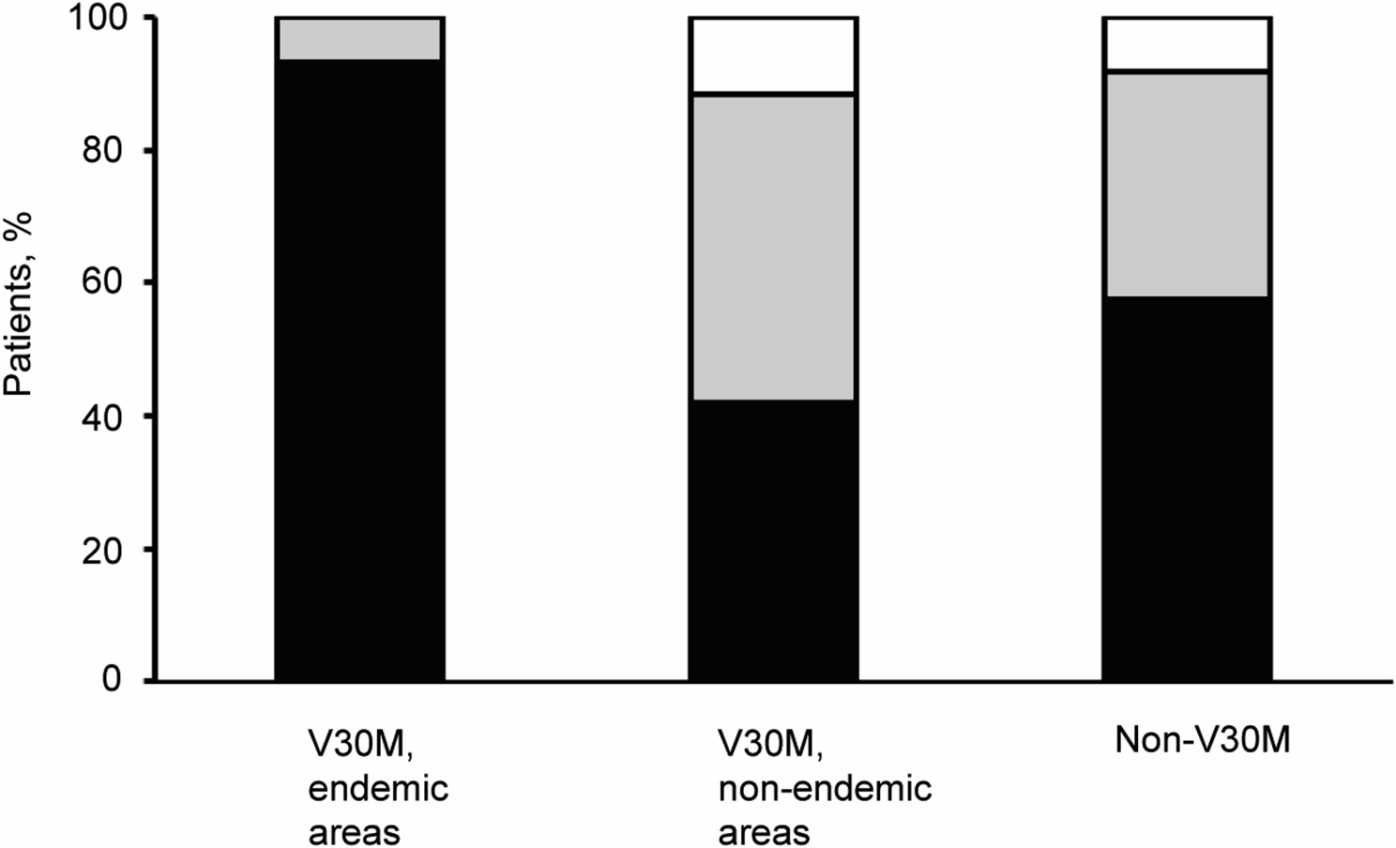
Family history patterns. The figure depicts the distribution of family history among the 235 patients with symptomatic ATTRv amyloidosis. Categories include confirmed family history (black boxes), absence of family history (gray boxes), and unknown family history (white boxes), highlighting hereditary trends across different genetic mutations

The onset age of ATTRv amyloidosis showed significant variation: 42.4 ± 15.6 years for endemic V30M, 64.5 ± 11.9 years for non-endemic V30M, and 60.6 ± 14.6 years for non-V30M patients. Gender distribution differed across groups, with a female predominance in endemic V30M (0.6:1) and male predominance in non-endemic V30M (3.2:1) and non-V30M (1.9:1) (Table 1). Family history was present in 93.3% of endemic V30M cases but was less frequent in non-endemic V30M (42.0%) and non-V30M (57.5%) cases (Fig. 2 and Table 1).

**Table 1:** Demographic and clinical characteristics of ATTRv amyloidosis

	V30M, endemic	V30M, non-endemic	Non-V30M
Patients, n (%)	18 (7.7%)	114 (48.5%)	103 (43.8%)
Age, mean ± SD (years)	46.1 ± 14.5	68.9 ± 11.3	61.2 ± 13.6
Sex, n (%)			
Male	7 (38.9%)	87 (76.3%)	67 (65%)
Female	11 (61.1%)	27 (23.7%)	36 (35%)
Family history, %	93.3%	42%	57.5%
Onset age, mean ± SD (years)	42.4 ± 15.6	64.5 ± 11.9	60.6 ± 14.6
Time from onset to diagnosis, mean ± SD (years)	2.7 ± 1.7	4.6 ± 4.5	3.6 ± 3.7

This table presents the demographic and clinical characteristics of symptomatic ATTRv amyloidosis, classified by TTR mutation type

**Fig. 3:**
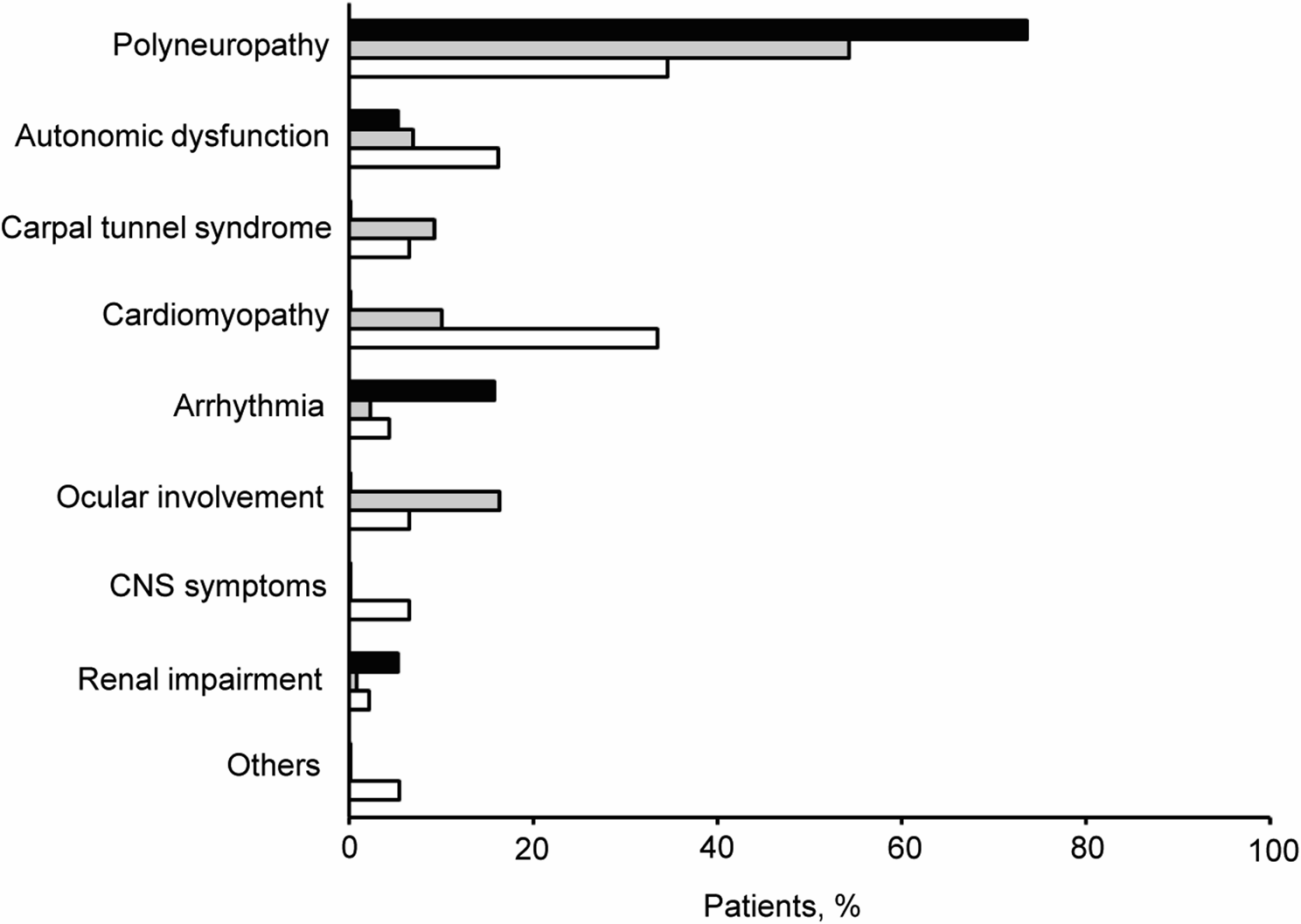
Clinical presentation of initial symptoms. This figure shows the initial symptoms observed in the 235 patients with symptomatic ATTRv amyloidosis. Patients are categorized based on their genetic mutations as follows: V30M in endemic areas (black boxes), V30M in non-endemic areas (gray boxes), and non-V30M mutations (white boxes)

Figure 3 depicts the distribution of initial symptoms among patients with symptomatic ATTRv amyloidosis, classified by *TTR* mutation type. Polyneuropathy was the most common symptom observed in 73.7% of endemic V30M cases, 54.3% of non-endemic V30M cases, and 34.6% of non-V30M cases. Secondary initial symptoms differed significantly among the mutation groups, with arrhythmia being more frequent in endemic V30M cases, ocular involvement in non-endemic V30M cases, and cardiomyopathy in non-V30M cases. Additionally, Table 1 presents the association between each genetic mutation and its corresponding clinical symptoms. Our cohort included neuropathy-dominant, cardiomyopathy-dominant, mixed, and oculoleptomeningeal-dominant phenotypes. These findings elucidate the clinical heterogeneity associated with specific mutations (Fig. 3**and** Table 2).

**Table 2:** Association between genotype and phenotype

genotype	Number of patients	phenotype
V30M, non- endemic	114	polyneuropathy (main), ocular involvement, cardiomyopathy, carpal tunnel syndrome, autonomic dysfunction, arrhythmia, renal impairment
V30M, endemic	18	polyneuropathy (main), arrhythmia, autonomic dysfunction, renal impairment
S50I	8	cardiomyopathy, polyneuropathy, others
T60A	8	cardiomyopathy, polyneuropathy, arrhythmia, renal impairment
E61K	7	cardiomyopathy, polyneuropathy, autonomic dysfunction
I107V	7	polyneuropathy, carpal tunnel syndrome
Y114C	6	polyneuropathy, autonomic dysfunction, ocular involvement, arrhythmia
P24S	5	cardiomyopathy
G47R	5	autonomic dysfunction
A25T	4	CNS symptoms
Y114S	4	polyneuropathy, cardiomyopathy, carpal tunnel syndrome. others
G47V	3	polyneuropathy, autonomic dysfunction
S50R	3	autonomic dysfunction, others
V122I	3	cardiomyopathy, arrhythmia
V30A	2	ocular involvement
V30M/ K80R	2	cardiomyopathy
A45V	2	polyneuropathy
T49I	2	cardiomyopathy, polyneuropathy
T49S	2	cardiomyopathy, carpal tunnel syndrome
V94G	2	autonomic dysfunction
A97G	2	polyneuropathy
A19D	1	cardiomyopathy
V25S	1	polyneuropathy
V28M	1	polyneuropathy
V28S	1	autonomic dysfunction
F33V	1	ocular involvement
A36D	1	cardiomyopathy
D38A	1	polyneuropathy
P43S	1	cardiomyopathy
A45D	1	autonomic dysfunction
G53E	1	CNS symptoms
E54L	1	cardiomyopathy
L55P	1	others
H56R	1	carpal tunnel syndrome
Y69H	1	CNS symptoms
S77Y	1	polyneuropathy
W79L	1	cardiomyopathy
K80R	1	autonomic dysfunction
G83R	1	ocular involvement
E89D	1	cardiomyopathy
E89K	1	cardiomyopathy
E89Q	1	cardiomyopathy
R104H	1	renal impairment
Y105H	1	polyneuropathy
Y114H	1	polyneuropathy
A120S	1	carpal tunnel syndrome
V121A	1	others

This table presents information on the genotype, number of patients, and phenotype

**Fig. 4:**
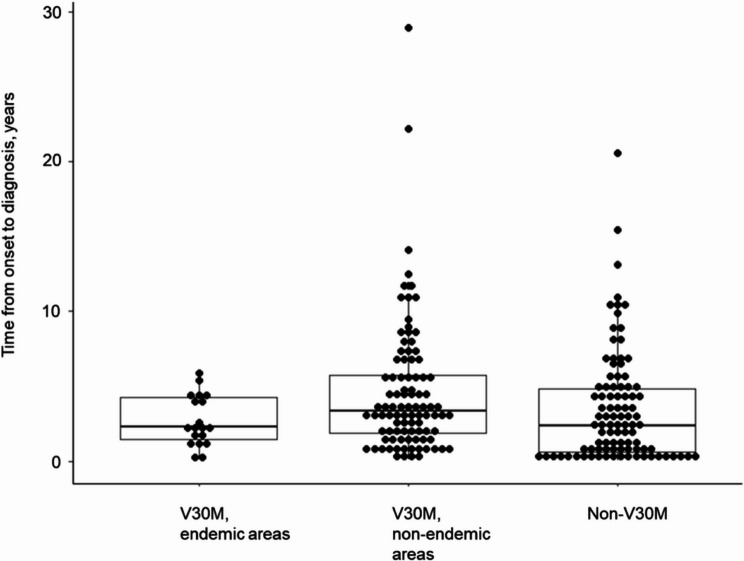
Variability in onset to diagnosis intervals. The time from symptom onset to diagnosis is presented for 235 symptomatic ATTRv amyloidosis patients stratified by the *TTR* mutation type. Categories include V30M mutations in endemic and non-endemic areas and non-V30M mutations, highlighting diagnostic delays among subgroups

The mean time from symptom onset to diagnosis in patients with ATTRv amyloidosis was also analyzed and stratified according to the mutation type. Patients with endemic V30M mutations experienced the shortest diagnostic delay (2.7 ± 1.7 years), whereas those with non-endemic V30M mutations and non-V30M mutations exhibited longer intervals (4.6 ± 4.5 years and 3.6 ± 3.7 years, respectively), reflecting differences in clinical presentation and diagnostic challenges (Fig. 4**and** Table 1).

**Fig. 5:**
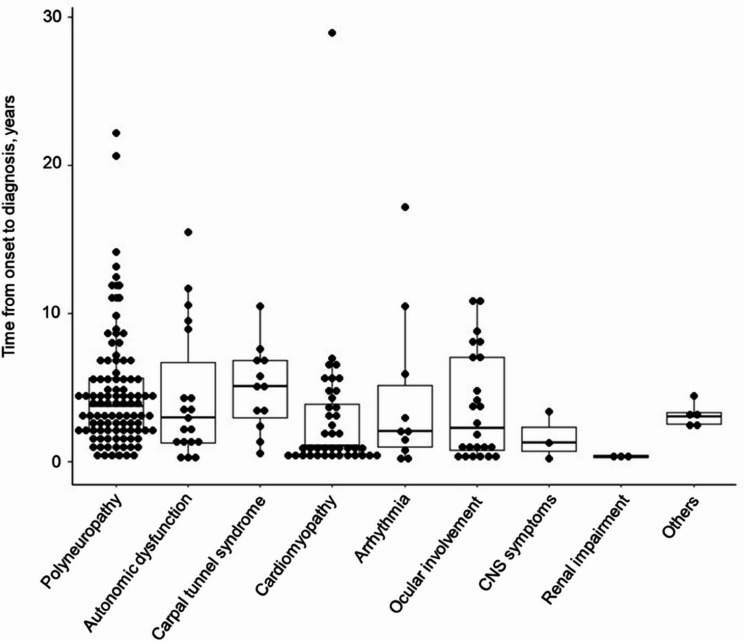
Impact of initial symptoms on diagnosis timing. This figure analyzes the diagnostic intervals in 235 symptomatic patients with ATTRv amyloidosis, based on their initial symptoms. Categories include carpal tunnel syndrome, polyneuropathy, and other symptoms, showing the influence of clinical presentation on diagnostic delays

Figure 5 presents the mean diagnostic delays for patients with ATTRv amyloidosis stratified by initial symptoms. The shortest time to diagnosis was observed in patients with renal impairment (0.4 ± 0.1 years), whereas patients presenting with carpal tunnel syndrome experienced the longest delay (4.9 ± 2.8 years). Other notable delays included 4.6 ± 3.9 years for polyneuropathy and 2.9 ± 4.7 years for cardiomyopathy (Fig. 5).

Histopathological analyses identified the gastrointestinal tract (28.6%, *n* = 60), heart (25.2%, *n* = 53), and abdominal fat (19.5%, *n* = 41) as the most common biopsy sites. Most sites demonstrated high positivity rates, with the nerves, vitreous body, and ligamentum carpi transversum achieving 100% positivity. Detection rates of amyloid deposits were similarly high in the gastrointestinal tract (88%), heart (98%), and abdominal fat (95%), reflecting the diagnostic utility of these biopsy sites (Table 3).

**Table 3:** Biopsy sites and amyloid deposit positivity rates

Biopsy sites	Number	Positive	Negative	Positive rate (%)
Gastrointestinal tract	60	53	7	88
Heart	53	52	1	98
Abdominal fat	41	39	2	95
Nerve	14	14	0	100
Vitreous body	13	13	0	100
Skin	13	12	1	92
Ligamentum carpi transversum	4	4	0	100
Meninges	3	3	0	100
Kidney	3	2	1	67
Prostate	2	2	0	100
Lip	1	1	0	100
Salivary glands	1	1	0	100
Ligamentum flavum	1	1	0	100
Muscle	1	1	0	100

This table presents the distribution of the positivity rates for amyloid deposits across various biopsy sites

Among the 57 patients diagnosed with ATTRv amyloidosis from 2020 to 2023, serum M-protein was measured in 30 patients and serum FLCs was measured in 28 patients.

Monoclonal protein with lambda light chain and a reduced FLC κ/λ ratio (0.15) was identified in one patient with Y114H. In addition, 99mTc-PYP scintigraphy was performed in 24 patients. Among them, 23 patients showed 99mTc-PYP-positive uptake in the heart, while one patient with Y114C showed 99mTc-PYP-negative uptake in the heart.

## Discussion

This retrospective, single-center study revealed that patients with V30M mutations in non-endemic areas and non-V30M mutations were more common than previously reported. Clinical characteristics differed between V30M patients in endemic and non-endemic areas as well as in non-V30M patients. Endemic V30M cases were diagnosed earlier because of the higher prevalence of family history and periodic monitoring. In contrast, non-endemic V30M and non-V30M patients, often lacking a family history, were frequently misdiagnosed. Additionally, patients with carpal tunnel syndrome (CTS) or polyneuropathy at onset experienced longer diagnostic delays. Understanding these differences is crucial for early diagnosis and timely intervention, particularly, given the availability of disease-modifying therapies.

ATTRv amyloidosis, previously thought to be confined to endemic regions, is now known to affect both endemic and non-endemic areas worldwide [2–7]. In this study, 46 *TTR* mutations were detected in 235 symptomatic patients, compared to 25 mutations identified in 104 patients over a 5-year period in our earlier study. These findings highlight the increasing potential of diagnosing additional cases in Japan [6].

Clinical characteristics varied among patients with V30M mutations in endemic and non-endemic areas and those with non-V30M mutations. Endemic V30M cases exhibited early-onset, high penetrance, and female predominance, whereas non-endemic V30M cases showed late-onset, low penetrance, and male predominance. Non-V30M cases presented with diverse symptoms, late-onset, low penetrance, and male predominance. The causes of these differences remain unclear but are likely influenced by multiple factors. First, the geographic origin of the *TTR* V30M mutation may affect disease onset and penetrance, with early onset and high penetrance observed in Portugal and Japanese endemic regions, and late onset and low penetrance seen in Sweden and non-endemic areas of Japan [6, 8, 20, 21]. Second, mutations in non-coding regions of the TTR gene may regulate gene expression and influence both the age at onset and the clinical phenotype spectrum. Third, sex differences may contribute to phenotypic variation, as cardiac involvement is more frequently observed in male patients with non-endemic V30M or non-V30M mutations. Additionally, maternal inheritance may be associated with an earlier age at onset and higher disease penetrance [22].

Possible explanations include differences in pathology: early-onset cases exhibit small-fiber and neuronal cell loss with amyloid deposition in the sympathetic ganglia, whereas late-onset cases show preserved unmyelinated fibers and amyloid deposition in the dorsal root ganglia [20]. Phenotypic differences may also be related to TTR fragmentation; late-onset cases contain C-terminal TTR fragments in amyloid deposits, whereas early-onset cases have only full-length TTR [23]. Additionally, the stability of variant TTR and ERAD mechanisms play a role, as destabilized variants such as A25T can bypass ERAD with T4 chaperoning, leading to CNS-selective amyloidosis [24].

Patients with ATTR V30M amyloidosis in endemic areas were diagnosed earlier than those in non-endemic areas and patients with non-V30M amyloidosis, consistent with findings from a global survey [25]. Early diagnosis in endemic areas is attributed to the high prevalence of family history and regular monitoring of at-risk family members, which facilitate timely detection [26]. In contrast, patients with non-endemic V30M and non-V30M rarely have a family history, complicating early diagnosis. These cases are frequently misdiagnosed as CIDP, diabetic neuropathy, or lumbar canal stenosis, highlighting the need for increased clinical awareness to improve diagnostic accuracy [27, 28].

This study has few limitations. It relied on retrospective data from a single referral center with a limited number of patients. Moreover, the cohort included more cases of V30M in non-endemic areas and non-V30M mutations than V30M mutations in endemic areas, reflecting higher referral rates from specialists in non-endemic regions.

In conclusion, this study highlights the genetic and clinical diversity of patients with ATTRv amyloidosis. Patients with V30M in non-endemic areas and non-V30M mutations were more prevalent than expected, with observed variations in clinical features and diagnostic timelines based on the mutation type and initial symptoms.

## Data Availability

All data generated or analysed during this study are included in this published article.

## References

[1] Ando Y, Adams D, Benson MD, et al. Guidelines and new directions in the therapy and monitoring of ATTRv amyloidosis. Amyloid. 2022;29:143–55. 35652823 10.1080/13506129.2022.2052838

[2] Andrade C. A peculiar form of peripheral neuropathy; familiar atypical generalized amyloidosis with special involvement of the peripheral nerves. Brain. 1952;75(3):408–27. 12978172 10.1093/brain/75.3.408

[3] Araki S, Mawatari S, Ohta M, et al. Polyneuritic amyloidosis in a Japanese family. Arch Neurol. 1968;18(6):593–602. 5652991 10.1001/archneur.1968.00470360015001

[4] Andersson R. Familial amyloidosis with polyneuropathy. A clinical study based on patients living in Northern Sweden. Acta Med Scand Suppl. 1976;590:1–64. 1064291

[5] Tawara S, Nakazato M, Kangawa K, et al. Identification of amyloid prealbumin variant in Familial amyloidotic polyneuropathy (Japanese type). Biochem Biophys Res Commun. 1983;116(3):880–8. 6651852 10.1016/s0006-291x(83)80224-1

[6] Yamashita T, Ueda M, Misumi Y, et al. Genetic and clinical characteristics of hereditary transthyretin amyloidosis in endemic and non-endemic areas: experience from a single-referral center in Japan. J Neurol. 2018;265(1):134–40. 29177547 10.1007/s00415-017-8640-7

[7] Mutations in transthyretin gene (TTR). [Mutations in hereditary amyloidosis website]. Available at: http://amyloidosismutations.com/mut-attr.php. Accessed November 27, 2024.

[8] Koike H, Misu K, Ikeda S, et al. Study group for hereditary neuropathy in japan. Type I (transthyretin Met30) Familial amyloid polyneuropathy in japan. early- vs late-onset form. Arch Neurol. 2002;59(11):1771–6. 12433265 10.1001/archneur.59.11.1771

[9] Conceição I, De Carvalho M. Clinical variability in type I Familial amyloid polyneuropathy (Val30Met): comparison between late- and early-onset cases in Portugal. Muscle Nerve. 2007;35(1):116–8. 16969832 10.1002/mus.20644

[10] Ueda M, Ando Y. Recent advances in transthyretin amyloidosis therapy. Transl Neurodegener. 2014;3:19. 25228988 10.1186/2047-9158-3-19 PMC4165622

[11] Benson MD. Liver transplantation and transthyretin amyloidosis. Muscle Nerve. 2013;47(2):157–62. 23169427 10.1002/mus.23521

[12] Yamashita T, Ando Y, Okamoto S, et al. Long-term survival after liver transplantation in patients with Familial amyloid polyneuropathy. Neurology. 2012;78(9):637–43. 22345221 10.1212/WNL.0b013e318248df18

[13] Coelho T, Maia LF, Martins da Silva A, et al. Tafamidis for transthyretin Familial amyloid polyneuropathy: a randomized, controlled trial. Neurology. 2012;79(8):785–92. 22843282 10.1212/WNL.0b013e3182661eb1 PMC4098875

[14] Berk JL, Suhr OB, Obici L, et al. Diflunisal trial consortium. Repurposing diflunisal for Familial amyloid polyneuropathy: a randomized clinical trial. JAMA. 2013;310(24):2658–67. 24368466 10.1001/jama.2013.283815 PMC4139164

[15] Adams D, Gonzalez-Duarte A, O’Riordan WD, et al. Patisiran, an RNAi therapeutic, for hereditary transthyretin amyloidosis. N Engl J Med. 2018;379(1):11–21. 29972753 10.1056/NEJMoa1716153

[16] Adams D, Tournev IL, Taylor MS, et al. HELIOS-A collaborators. Efficacy and safety of Vutrisiran for patients with hereditary transthyretin-mediated amyloidosis with polyneuropathy: a randomized clinical trial. Amyloid. 2023;30(1):1–9. 35875890 10.1080/13506129.2022.2091985

[17] Benson MD, Waddington-Cruz M, Berk JL, et al. Inotersen treatment for patients with hereditary transthyretin amyloidosis. N Engl J Med. 2018;379(1):22–31. 29972757 10.1056/NEJMoa1716793 PMC12611561

[18] Dorbala S, Ando Y, Bokhari S, et al. ASNC/AHA/ASE/EANM/ HFSA/ISA/SCMR/SNMMI expert consensus recommendations for multimodality imaging in cardiac amyloidosis: part 1 of 2-evidence base and standardized methods of imaging. J Nucl Cardiol. 2019;26:2065–123. 31468376 10.1007/s12350-019-01760-6

[19] Naiki H, Sekijima Y, Ueda M, et al. Human amyloidosis, still intractable but becoming curable: the essential role of pathological diagnosis in the selection of type-specific therapeutics. Pathol Int. 2020;70(4):191–8. 31961039 10.1111/pin.12902

[20] Koike H, Misu K, Sugiura M, et al. Pathology of early- vs late-onset TTR Met30 Familial amyloid polyneuropathy. Neurology. 2004;63(1):129–38. 15249622 10.1212/01.wnl.0000132966.36437.12

[21] Ohmori H, Ando Y, Makita Y, et al. Common origin of the Val30Met mutation responsible for the amyloidogenic transthyretin type of Familial amyloidotic polyneuropathy. J Med Genet. 2004;41(4):e51. 15060127 10.1136/jmg.2003.014803 PMC1735751

[22] Carvalho E, Dias A, Coelho T, et al. Hereditary transthyretin amyloidosis: a myriad of factors that influence phenotypic variability. J Neurol. 2024;271(9):5746–61. 38907862 10.1007/s00415-024-12509-8 PMC11377651

[23] Ueda M. Transthyretin: its function and amyloid formation. Neurochem Int. 2022;155:105313. 35218869 10.1016/j.neuint.2022.105313

[24] Sekijima Y, Wiseman RL, Matteson J, et al. The biological and chemical basis for tissue-selective amyloid disease. Cell. 2005;121(1):73–85. 15820680 10.1016/j.cell.2005.01.018

[25] Dispenzieri A, Coelho T, Conceição I, et al. THAOS investigators. Clinical and genetic profile of patients enrolled in the transthyretin amyloidosis outcomes survey (THAOS): 14-year update. Orphanet J Rare Dis. 2022;18(1):236. 10.1186/s13023-022-02359-w PMC9206752 35717381

[26] Ueda M, Sekijima Y, Koike H, et al. Monitoring of asymptomatic family members at risk of hereditary transthyretin amyloidosis for early intervention with disease-modifying therapies. J Neurol Sci. 2020;414:116813. 32353608 10.1016/j.jns.2020.116813

[27] Planté-Bordeneuve V, Ferreira A, Lalu T, et al. Diagnostic pitfalls in sporadic transthyretin Familial amyloid polyneuropathy (TTR-FAP). Neurology. 2007;69(7):693–8. 17698792 10.1212/01.wnl.0000267338.45673.f4

[28] Cortese A, Vegezzi E, Lozza A, et al. Diagnostic challenges in hereditary transthyretin amyloidosis with polyneuropathy: avoiding misdiagnosis of a treatable hereditary neuropathy. J Neurol Neurosurg Psychiatry. 2017;88(5):457–8. 28188196 10.1136/jnnp-2016-315262 PMC5529976

